# Unraveling the Causal Linkages of *RBP7* and *SCGB3A1* on Pelvic Organ Prolapse: Multifaceted Insights From Genome‐Wide Mendelian Randomization, Single‐Cell RNA Analysis, and Network Pharmacology

**DOI:** 10.1155/bmri/9785848

**Published:** 2026-01-04

**Authors:** Ying Yang, Weiyuan Xing, Xiaoqin Wang, Qiran Sun, Ningning Hu, Liwen Zhang, Fuyun Dong, Rujun Chen

**Affiliations:** ^1^ Department of Obstetrics and Gynecology, Shanghai Fifth People′s Hospital, Fudan University, Shanghai, China, fudan.edu.cn; ^2^ Center of Community-Based Health Research, Fudan University, Shanghai, China, fudan.edu.cn

**Keywords:** mendelian randomization, pelvic organ prolapse, RBP7, SCGB3A1, single-cell sequencing, therapeutic drugs

## Abstract

**Background:**

Pelvic organ prolapse (POP) is a common pelvic floor disorder in middle‐aged and elderly women. Its pathophysiology is complex, involving weakened pelvic floor muscles and connective tissues. There is a need to explore its underlying pathogenesis and develop effective treatments.

**Methods:**

We integrated single‐cell sequencing (scRNA‐seq) data analysis with Mendelian randomization (MR) analysis. scRNA‐seq data of vaginal mucosal tissue were obtained from individuals with and without pelvic organ prolapse (POP); we then performed dimensionality reduction and cell subset identification. MR was conducted using GWAS summary statistics and eQTL data, following STROBE‐MR guidelines. We also performed protein‐protein interaction analysis, functional enrichment analysis, drug prediction, and molecular docking.

**Results:**

We identified *RBP7* as a POP risk factor and *SCGB3A1* as a protective factor. *RBP7* high expression increased POP risk (IVW, OR 1.262, 95% CI 1.093–1.459, *p* = 0.002), whereas *SCGB3A1* high expression decreased it (IVW, OR 0.907, 95% CI 0.844–0.975, *p* = 0.008). We found associated key genes and their biological processes and signaling pathways. We also predicted potential drugs and their binding affinities.

**Conclusion:**

The study highlights the significance of fibroblast gene expression changes in collagen metabolism for POP. It identified risk and protective genes and explored potential drugs. Future research should verify *SCGB3A1* functions in fibroblasts, conduct preclinical drug trials, and clarify POP molecular mechanisms.

## 1. Introduction

Pelvic organ prolapse (POP), a prevalent pelvic floor dysfunction, notably impacts the health of middle‐aged and elderly women, emerging as a major concern especially within the female demographic [1]. This intricate, multifactorial condition has profound implications for patients′ quality of life, healthcare resource use, and overall welfare. POP occurs when pelvic organs like the uterus, vagina, bladder, and rectum shift from their normal anatomical sites, slide down along the vagina, and may protrude through the vaginal orifice, greatly inconveniencing patients and affecting their physical and mental health [2]. POP typically presents with a range of symptoms and traits, including, yet not restricted to, pelvic organ displacement, urinary and fecal incontinence, and a sensation of pelvic pressure or fullness [1]. Some individuals also experience symptoms such as anal bulging and fecal leakage. Documented risk factors encompass vaginal delivery, number of pregnancies, forceps‐assisted delivery, increasing age, postmenopause, connective tissue diseases, obesity, and chronic constipation [3].

The pathophysiology underpinning POP is intricate, stemming from a confluence of diverse factors. The weakening of pelvic floor muscles and connective tissues, triggered by childbirth, the aging process, hormonal shifts, obesity, chronic surges in intra‐abdominal pressure (due to chronic cough or constipation), and genetic predisposition, all contribute significantly to POP development [3]. Grasping these risk factors is essential for devising effective prevention and treatment strategies. However, there is an urgent need to explore the underlying pathogenic mechanisms tied to these risk factors. This exploration will not just enhance our understanding of the disease course but may also uncover new therapeutic targets and more refined preventive measures. Thus, an in‐depth investigation into the pathophysiological changes and molecular mechanisms in POP is desperately needed.

The Mendelian randomization (MR) method makes use of single nucleotide polymorphisms (SNPs) as instrumental variables (IVs) to gauge possible causal associations between exposures and outcomes [4–7]. Through the utilization of GVs to mimic the random assignment of traits passed from parents to their offspring, MR efficiently overcomes problems associated with confounding and reverse causality [8]. When the crucial assumptions are met, MR investigations are capable of facilitating causal inference while minimizing environmental confounding and reverse causation.

Here, we performed a MR research, making use of single‐cell sequencing data, expression quantitative trait locus (eQTL) data [9], and genome‐wide association study (GWAS) data. We intended to identify the crucial genes that are causally related to the occurrence of POP.

## 2. Methods

### 2.1. Study Design

Firstly, we acquired the single‐cell sequencing data that was provided by a single‐cell RNA sequencing (scRNA‐seq) study concerning POP [10]. This dataset encompassed the sequencing information of vaginal mucosal tissue from POP patients as well as that of normal vaginal mucosal tissue from individuals without POP. Subsequently, we carried out dimensionality reduction analysis and the identification of cell subsets on this data and successfully obtained the transcriptome dataset of fibroblasts. After that, we pinpointed the differentially expressed genes in fibroblasts between the POP group and the normal group. These differentially expressed genes were designated as candidate genes for the subsequent research.

**Figure 1:**
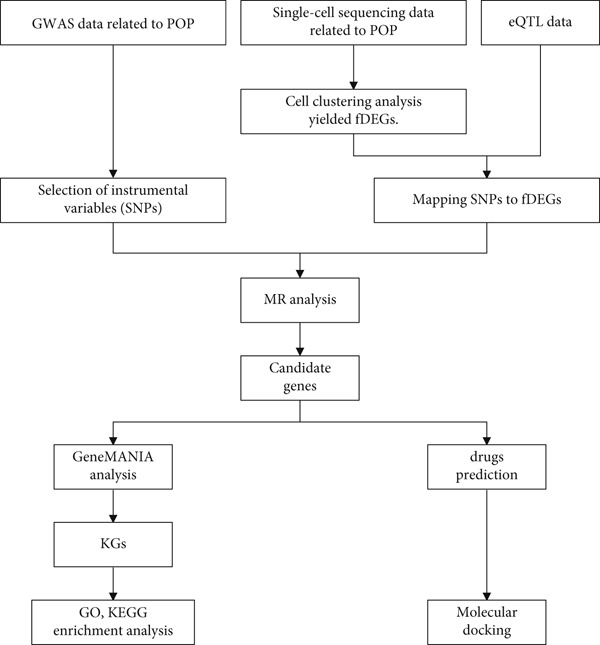
The flowchart for this research.

Next, in accordance with the STROBE‐MR guidelines [11], we carried out a MR analysis. The MR approach is based on three assumptions: [1] The genetic variants functioning as IVs are linked to a specific protein; [2] These genetic variants have no connection with any unmeasured confounding factors related to POP; [3] The genetic variants are associated with POP solely through the specific protein and not via other pathways. Our analysis utilized publicly available GWAS summary statistics and eQTL data. Since no new data were collected, there was no requirement for additional ethical approval. The research process of this study is depicted in Figure 1.

In addition, we hereby declare that no large language model (LLM)‐based artificial intelligence generated content (AIGC) tools (such as ChatGPT) were used in the development of any portion of this manuscript.

### 2.2. Data Sources

The scRNA‐seq dataset relevant to POP is sourced from the GSE151192 dataset within the Gene Expression Omnibus (GEO) database, which can be accessed at https://www.ncbi.nlm.nih.gov/geo/query/acc.cgi?acc=GSE151192. This dataset includes the single‐cell sequencing data of the anterior vaginal wall tissue obtained from 16 patients suffering from anterior vaginal wall prolapse, as well as the single‐cell sequencing data of the normal vaginal wall tissue from five individuals without POP.

The summary‐level data regarding the associations between exposure‐associated SNPs and POP were retrieved from GWAS with the GWAS ID: finn‐b‐N14_FEMGENPROL, available at https://gwas.mrcieu.ac.uk/datasets/finn-b-N14_FEMGENPROL/. This GWAS involved 9092 patients diagnosed with POP and 68,969 controls during the discovery stage. Moreover, the eQTL data were publicly accessible from the IEU OpenGWAS project, which can be found at https://gwas.mrcieu.ac.uk/. Furthermore, we also incorporated a POP‐related GWAS dataset associated with East Asian individuals from Japan—including 771 cases and 76,625 controls—which served as the validation set [12].

### 2.3. scRNA‐Seq Data Analysis

The datasets′ quality control was carried out using the “Seurat” package [13] (Version 5.2.1) in R software (Version 4.3.2). At first, the “CreateSeuratObject” function was used to convert the samples into Seurat objects. Cells were selected with mitochondrial gene percentages lower than 25% and the count of unique genes ranging from 200 to 6000. The “NormalizeData” function was applied to normalize the data, and the “ScaleData” function was utilized to scale all genes. After that, principal component analysis (PCA) was conducted on the data. The “FindVariableFeatures” function was used to detect hypervariable genes, which were then applied for downstream analysis. The “FindClusters” function was employed to perform cell clustering and classification. Subsequently, the “SingleR” package [14] (Version 2.4.1) was used to match the single‐cell RNA‐seq data to a known reference dataset, and manual calibration was carried out to boost the accuracy and reliability of cell type annotation. Finally, the “FindAllMarkers” function was used to determine the differentially expressed genes between the fibroblasts in the POP group and those in the non‐POP group.

### 2.4. MR Statistical Analysis

We utilized the “TwoSampleMR” package [15] (Version 0.6.14) to carry out the MR analysis. The criteria for selecting IVs in the bidirectional MR were as follows: we screened for SNPs with a significant association, specifically those with a *p* value below 5 × 10^−8^, and set the threshold for removing linkage disequilibrium to an *r* ^2^ value of less than 0.001 [16]. We only selected SNPs with an effect allele frequency greater than 0.01, excluded palindromic SNPs and removed SNPs with an F‐statistic of less than 10 to prevent the bias associated with weak IVs [17]. For clear reproducibility of the work, we have prepared and shared all relevant data and code via GitHub (https://github.com/znxfdd/papercode2).

### 2.5. Mapping SNPs to Candidate Genes

We sourced the eQTL data of candidate genes from the IEU OpenGWAS project. This dataset consisted of genome‐wide SNPs linked to the expression‐level variations of these genes. Afterward, leveraging the previously mentioned results, we performed MR analysis on the IV SNPs and the exposure genes. To connect candidate genes to SNPs, we filtered SNPs according to the following criteria. In the MR test, the *p* value threshold for selecting pertinent eQTLs was set at 5 × 10^−8^. The eQTL‐selection region around the probe center had a 10,000 kb radius. Moreover, the threshold for removing LD was established as *r* ^2^ < 0.001.

### 2.6. GeneMANIA Analysis

In an effort to examine the relationships both among candidate genes and between them and other relevant genes, we made use of the Gene Multiple Association Network Integration Algorithm (GeneMANIA) tool [18] (http://www.genemania.org/). This tool was employed to explore protein–protein interactions. It works by identifying a group of genes or proteins that are highly probable to possess functional similarities to the selected genes or proteins based on their interactions. The tool assigns continuous weights in the range of 0–1, where these weights signify the degree of coregulation between genes. All the genes incorporated in the resulting protein–protein interaction map are designated as key genes, and for simplicity, we abbreviate them as KGs.

### 2.7. Functional Enrichment Analysis of KGs

In order to gain insights into the potential functions of KGs in the pathogenesis of POP, a series of functional enrichment analyses were carried out. The “clusterProfiler” R package [19] was applied to perform the enrichment analysis for Gene Ontology (GO), covering biological processes (BP), cellular components (CC), molecular functions (MF), as well as Kyoto Encyclopedia of Genes and Genomes (KEGG) pathways. A significance threshold of *p* < 0.05 was established. Subsequently, the most prominent GO terms and KEGG pathways were graphically presented using the “ggplot2” R package [20].

### 2.8. Potential Therapeutic Drugs Prediction

To obtain information on drug interactions and diseases related to each candidate gene, we searched the CTDbase database [21] (https://ctdbase.org/). After that, we embarked on an analysis of small‐molecule ligands that target these genes in the context of POP. For genes with a protective role, we concentrated on finding drugs that can enhance their expression levels. Conversely, for genes linked to an increased risk, our goal was to identify drugs that can suppress their expression.

### 2.9. Molecular Docking

We retrieved the two‐dimensional structures of each small‐molecule ligand drug from the PubChem database [22] (PDB, https://pubchem.ncbi.nlm.nih.gov/), imported them into Chem3D software to calculate the minimum free energy and convert them into three‐dimensional (3D) structures, sourced the 3D structures of the target proteins (receptors) from the RCSB Protein Data Bank [23] (RCSB PDB, https://www.rcsb.org/), imported these into PyMOL to remove water molecules and ligands, used the AutoDock tool (Version 1.5.6) to prepare the receptors and ligands by getting their PDBQT formats and creating a 3D grid box for the receptor for subsequent molecular docking simulations, carried out molecular docking analysis with AutoDock Vina (Version 1.1.2), and finally used PyMOL (https://www.pymol.org/) to visualize the best‐predicted binding site, with a binding energy of less than −5 kcal/mol considered as evidence of effective ligand‐receptor binding.

## 3. Results

### 3.1. Cell Clustering Based on Single‐Cell Sequencing Data and Identification of Fibroblast‐Related Differentially Expressed Genes in POP

First, we analyzed the single‐cell sequencing data of vaginal mucosa tissues from patients with POP and those of normal vaginal mucosa tissues from non‐POP individuals. The data were clustered into 26 subpopulations (Figure 2a). Subsequently, the “SingleR” package was utilized to annotate the cell types, and the cells were classified into stromal cells and fibroblasts (Figure 2b,c). In light of previous investigations, fibroblasts have been demonstrated to play a pivotal role in the pathogenesis and progression of POP [24, 25]. Consequently, we elected to focus on fibroblasts for subsequent research endeavors. Next, the differentially expressed genes in fibroblasts between the POP group and the non‐POP group were identified. These genes are likely to be involved in the pathogenesis of POP, and we named these genes fDEGs (Table S1).

Figure 2Dimensionality reduction, clustering, and cell type identification in single‐cell transcriptome sequencing. (a) Cell dimensionality reduction and clustering, (b) cell type identification, (c) T‐distributed stochastic neighbor embedding (tSNE) plots of the POP group and non‐POP group.

**Figure:**
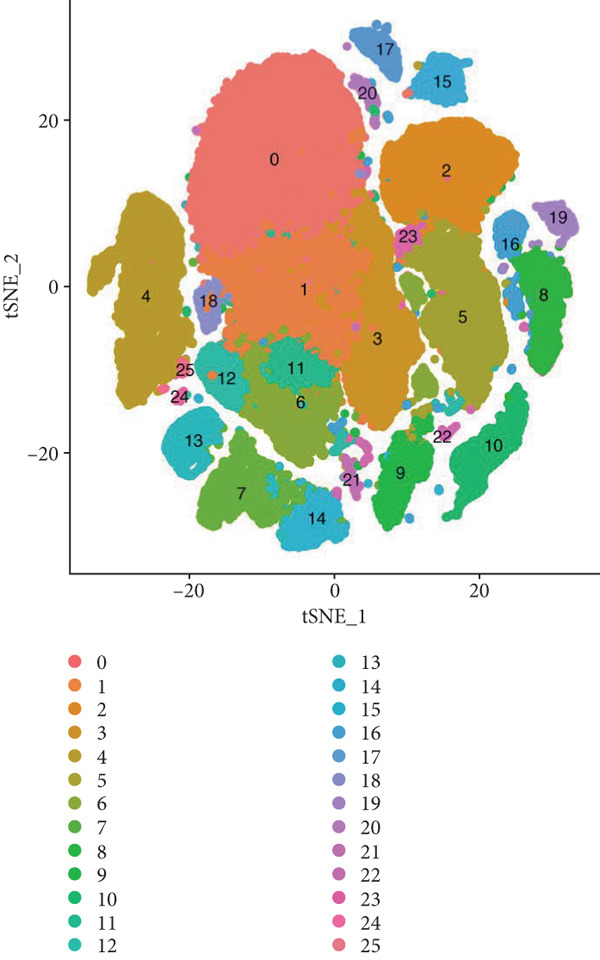
(a)

**Figure:**
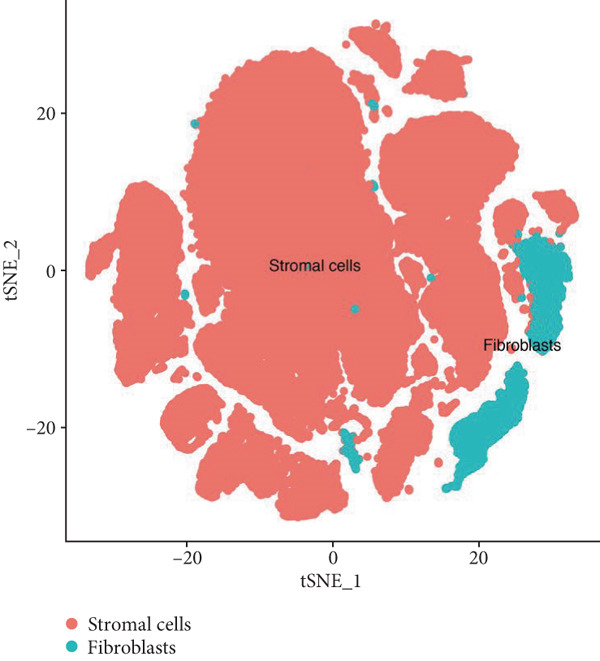
(b)

**Figure:**
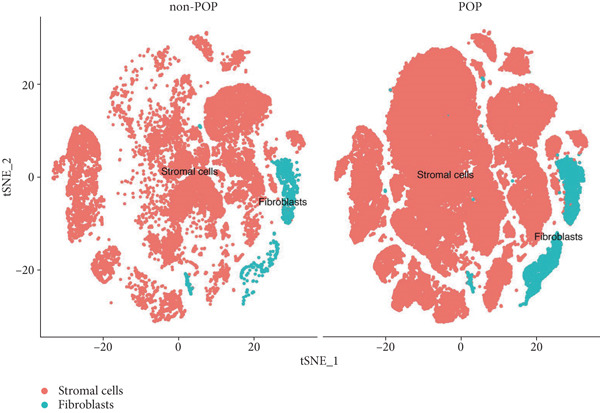
(c)

### 3.2. Identification of Candidate Genes With a Causal Link to POP

By leveraging eQTL data, we established associations between the fDEGs and SNPs. Subsequently, through conducting MR analysis, we were able to identify from the fDEGs the candidate genes with a causal relationship to POP, namely *RBP7* (*Retinol Binding Protein 7*) and *SCGB3A1* (*Secretoglobin Family 3A Member 1*) (Figure 3a,b). As illustrated in Figure 3a,b, *RBP7* was identified as a risk factor for POP, whereas *SCGB3A1* served as a protective factor for POP. High expression of *RBP7* was associated with an increased risk of POP (IVW, odds ratio [OR] 1.262 95% confidence interval [CI] 1.093–1.459, *p* = 0.002), while high expression of *SCGB3A1* was linked to a decreased risk of POP (IVW, OR 0.907, 95% CI 0.844–0.975, *p* = 0.008). The forest plot depicted in Figure 4a,b demonstrated the impact of SNPs associated with *RBP7* on the risk of POP. A funnel plot was constructed to evaluate the heterogeneity (Figure 4c), and a scatter plot illustrating the effects of SNPs on the exposure factor (*RBP7*) and the outcome (POP) is presented in Figure 4d. Furthermore, the forest plot shown in Figures 4e,f revealed the influence of SNPs related to *SCGB3A1* on the risk of POP. The funnel plot used for assessing the heterogeneity was presented in Figure 4g, and the scatter plot showing the effects of SNPs on the exposure factor (*SCGB3A1*) and the outcome (POP) is displayed in Figure 4h.

Figure 3Mendelian randomization analysis reveals the causal relationship between genes and the incidence of POP. (a) Forest plot illustrates *RBP7* and *SCGB3A1* genes′ influence on POP risk. (b) The volcano plot demonstrates the situation of genes associated with the incidence of POP.

**Figure:**
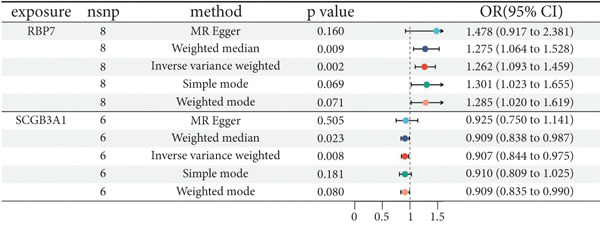
(a)

**Figure:**
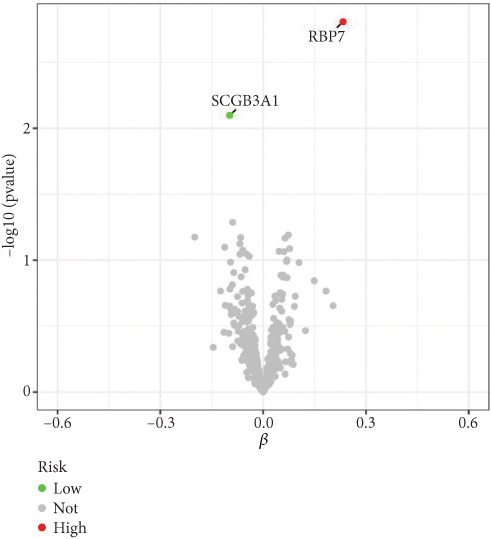
(b)

Figure 4The contributions of SNPs related to *RBP7* and *SCGB3A1* genes to the risk of POP. (a) The MR effect size of SNPs related to the *RBP7* gene on the risk of POP. (b) Leave‐one‐out sensitivity analysis of *RBP7* gene‐related SNPs on POP risk. (c) The funnel plot demonstrates the heterogeneity of *RBP7* gene‐related SNPs. (d) The scatter plot shows the relationship among SNPs, *RBP7*, and POP. (e) The MR effect size of SNPs related to the *SCGB3A1* gene on the risk of POP. (f) Leave‐one‐out sensitivity analysis of *SCGB3A1* gene‐related SNPs on POP risk. (g) The funnel plot demonstrates the heterogeneity of *SCGB3A1* gene‐related SNPs. (h) The scatter plot shows the relationship among SNPs, *SCGB3A1*, and POP.

**Figure:**
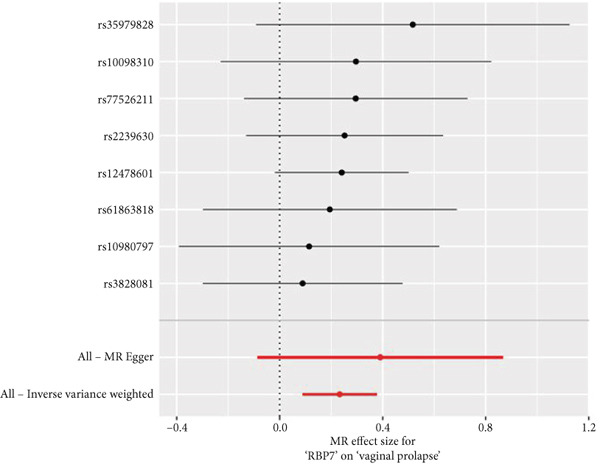
(a)

**Figure:**
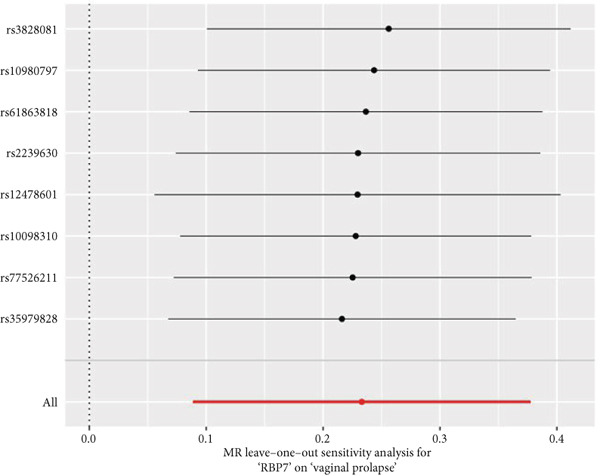
(b)

**Figure:**
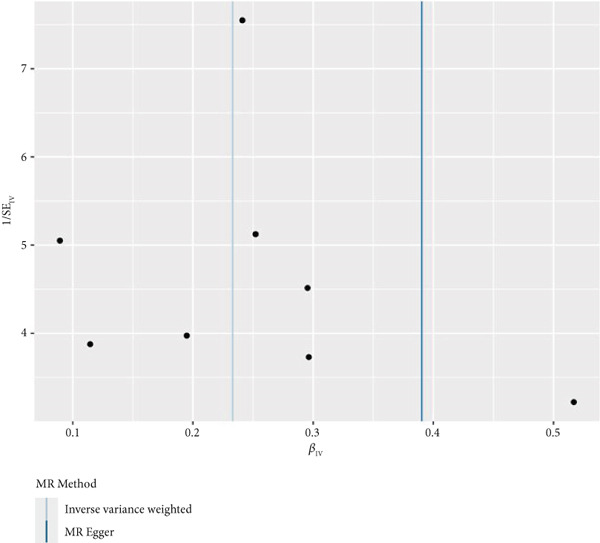
(c)

**Figure:**
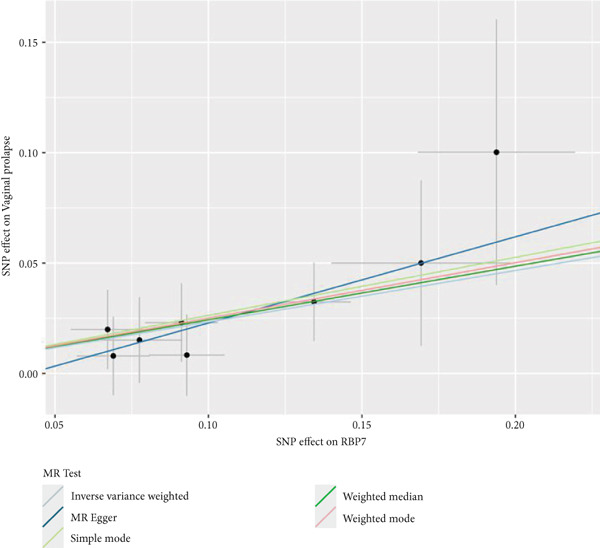
(d)

**Figure:**
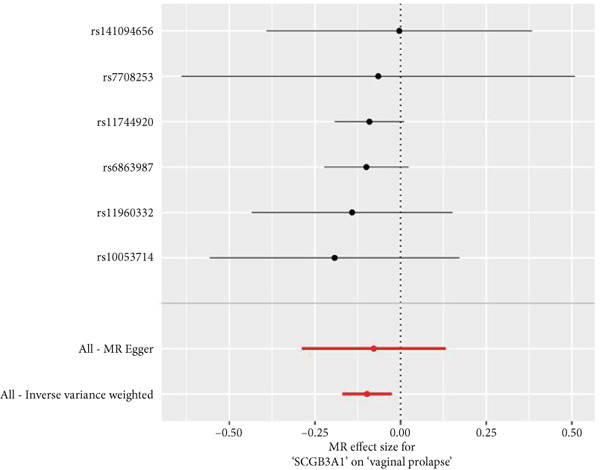
(e)

**Figure:**
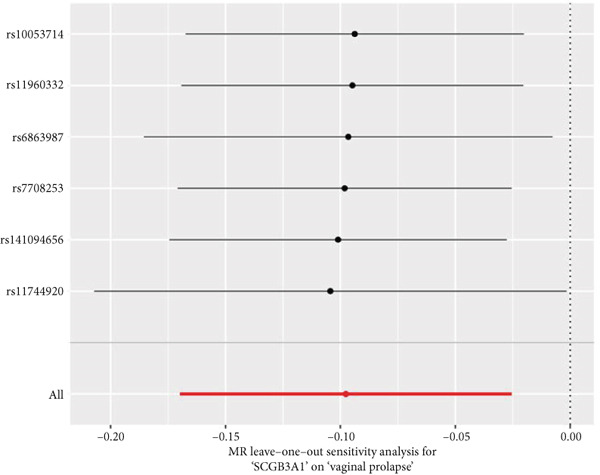
(f)

**Figure:**
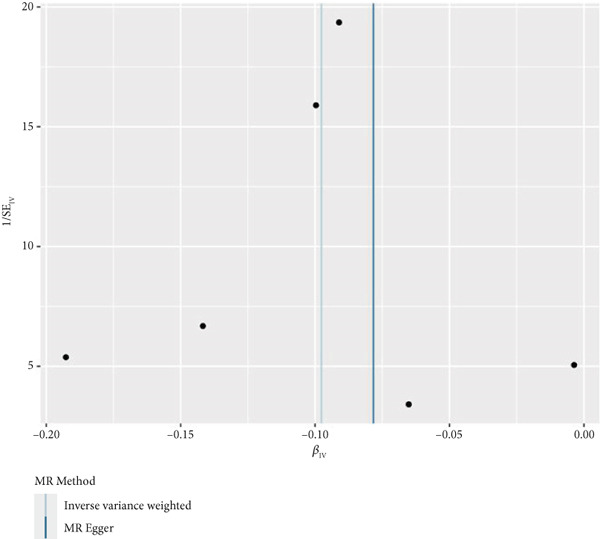
(g)

**Figure:**
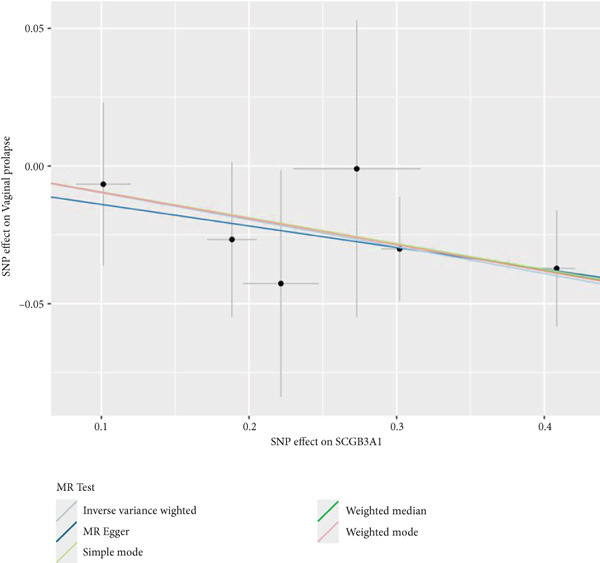
(h)

### 3.3. Protein–Protein Interaction Network of Candidate Genes

**Figure 5:**
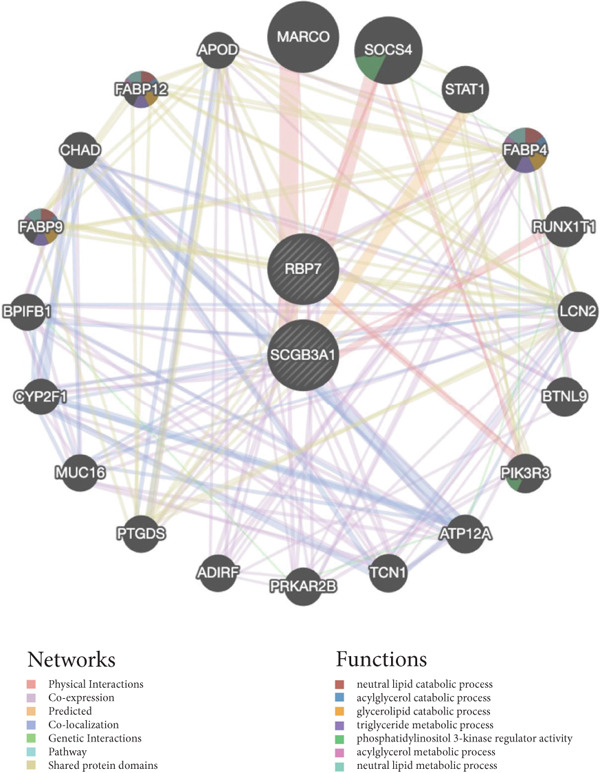
The protein‐protein interaction network of candidate genes.

To elucidate the roles that candidate genes (*RBP7* and *SCGB3A1*) play in the pathogenesis of POP, we employed the GeneMANIA tool to explore genes/proteins that exhibit the following associations with *RBP7* and *SCGB3A1*: physical interactions, coexpression, predicted, colocalization, genetic interactions, pathway, and shared protein domains (Figure 5). A total of interaction maps of 22 proteins were obtained and these genes were designated as KGs. Additionally, the results revealed that these KGs were involved in the following BP: neutral lipid catabolic process, acylglycerol catabolic process, glycerolipid catabolic process, triglyceride metabolic process, phosphatidylinositol 3‐kinase regulator activity, acylglycerol metabolic process, and neutral lipid metabolic process (Figure 5).

### 3.4. Functional Enrichment Analysis of KGs

To elucidate the roles of the proteins expressed by KGs in the pathogenesis of POP, we carried out a series of functional enrichment analyses on KGs. The outcomes of the GO analysis indicated that, with respect to BP, the top three significantly enriched functions were fatty acid transport, monocarboxylic acid transport, and lipid transport. In terms of CC, the top three significantly enriched components were specific granule lumen, specific granule, and phosphatidylinositol 3‐kinase complex. Regarding MF, the top three significantly enriched functions were fatty acid binding, monocarboxylic acid binding, and carboxylic acid binding (Figure 6a). The results of the KEGG analysis showed that the significantly enriched signaling pathways encompassed the JAK−STAT signaling pathway, the regulation of lipolysis in adipocytes, the C−type lectin receptor signaling pathway, and the Toll−like receptor signaling pathway (Figure 6b).

Figure 6The functional enrichment analysis of KGs. (a) The bar chart shows the results of the GO enrichment analysis. (b) The bar chart shows the results of the KEGG enrichment analysis.

**Figure:**
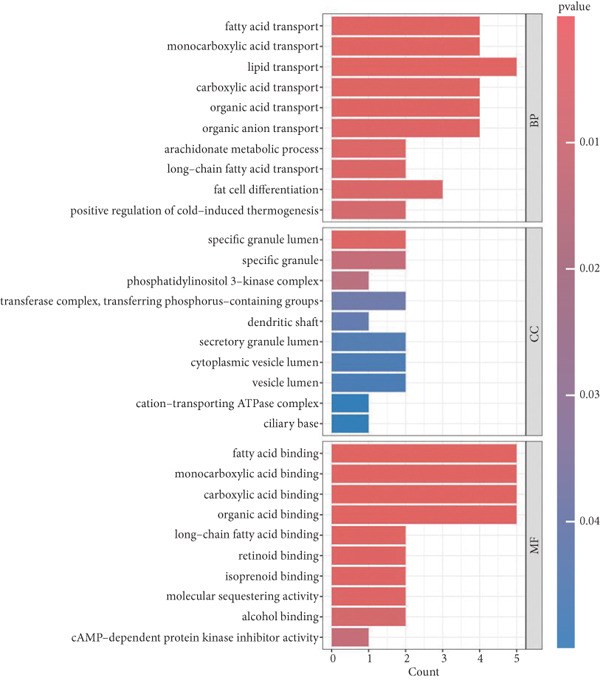
(a)

**Figure:**
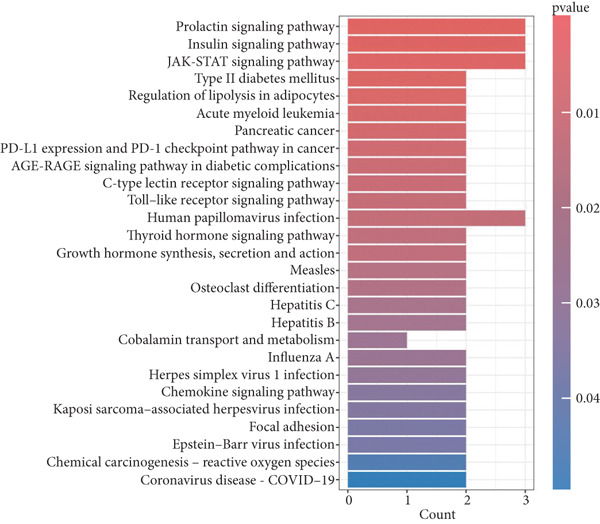
(b)

### 3.5. Drug Prediction and Molecular Docking

By retrieving the CTDbase database, we obtained a list of drugs that can regulate *RBP7* and *SCGB3A1*. As *RBP7* is a risk factor for POP, we classified the drugs that can reduce its expression level as the candidate therapeutic agents. Conversely, since *SCGB3A1* serves as a protective factor for POP, we designated the drugs capable of upregulating its expression level as candidate therapeutic drugs. Subsequently, to assess the binding probabilities of these candidate therapeutic drugs to RBP7 and SCGB3A1, we conducted a prediction of the drug binding affinity. The results are presented in Table S2 and Table S3. Finally, we selected the top five drugs with the highest binding affinities for molecular docking, which illustrated the ability of these drugs to bind to the target proteins (Figure 7a,b).

Figure 7Molecular docking prediction of therapeutic drugs related to candidate genes. (a).The simulated binding diagrams of benzo(a)pyrene, estradiol, fenretinide, diethylhexyl phthalate, and 3,4,5,3 ^′^,4 ^′^‐pentachlorobiphenyl with *RBP7*. (b).The simulated binding diagrams of benzo(k)fluoranthene, perfluorooctane sulfonic acid, triptonide, aflatoxin B1, and doxorubicin with *SCGB3A1*.

**Figure:**
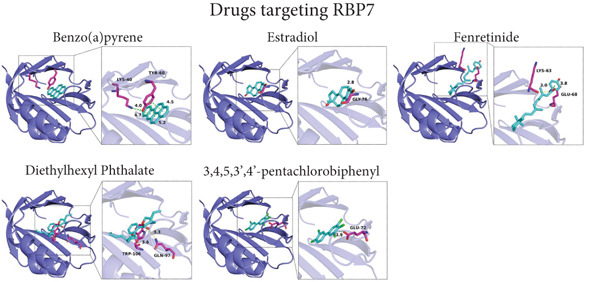
(a)

**Figure:**
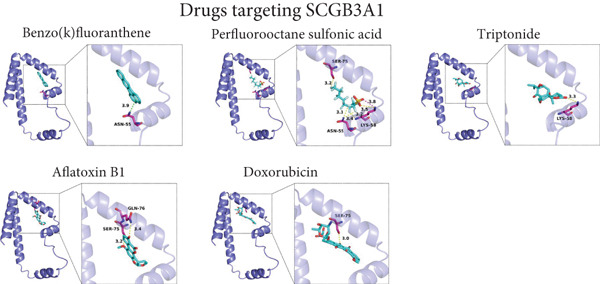
(b)

### 3.6. Validation in an East Asian Cohort (Japanese GWAS Dataset)

**Figure 8:**
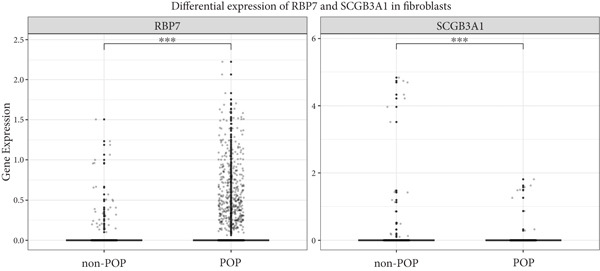
Differential expression of *RBP7* and *SCGB3A1* in fibroblasts. ∗∗∗*p* < 0.001.

To assess cross‐population generalizability of *RBP7* and *SCGB3A1* associations with POP, we validated it using a Japanese GWAS dataset (PMID: 39349682) [12], a well‐characterized East Asian POP cohort, to evaluate the reproducibility of European‐identified signals in non‐European populations. In this population, *RBP7* and *SCGB3A1* were differentially expressed in fibroblasts in the POP group compared with the non‐POP group (Figure 8).

For *RBP7*, located on Chromosome 1 with a start position of 9997054 and an end position of 10016021, 243 tag SNPs capturing variation across the gene and flanking regions were analyzed. Logistic regression identified 51 SNPs with nominal significance (*p* < 0.05) but discordant effect directions. This heterogeneity may reflect population‐specific LD patterns (tagged SNPs correlating with distinct causal variants in East Asians vs. Europeans) or gene–environment interactions (e.g., dietary vitamin A differences and what is a key *RBP7* regulator) modulating associations across ethnicities.

For *SCGB3A1*, located on Chromosome 5 with a start position of 180590105 and an end position of 180591499, 495 tag SNPs analyzed via the same framework were found to show no significant associations with POP (all *p* ≥ 0.05). This may result from reduced power in the Japanese cohort, population‐specific LD decay disrupting correlations with true causal variants, or ethnic‐specific epigenetic regulation diminishing SCGB3A1′s protective role in East Asian POP.

Forest plots displaying odds ratios (ORs) and 95% CIs for all SNPs (Figure S1) facilitate comparison of effect sizes, significance, and directional consistency. Manhattan plots mapping −log_10_(*p*) values across *RBP7* and *SCGB3A1* regions (Figure S2) illustrate signal distribution and highlight *RBP7* nominal significance clusters. These visualizations confirm *RBP7* effect direction inconsistency and the absence of significant *SCGB3A1* signals in the Japanese cohort.

## 4. Discussion

In this study, we innovatively integrated single‐cell sequencing data analysis with MR analysis. Through this combined approach, we identified the differentially expressed genes in fibroblasts that play a crucial role in the pathogenesis of POP. By utilizing eQTL data, we associated the candidate genes with SNPs and ultimately pinpointed the KGs that have a causal relationship with the onset of POP. This research provides a significant scientific foundation for exploring the pathogenesis of POP and discovering potential therapeutic targets.

Fibroblasts hold a central position in the development of POP due to their essential function in safeguarding the connective tissue′s integrity [26]. In cases of POP, the quantity and quality of fibroblasts often deteriorate, which exerts substantial influence on the condition′s pathophysiological mechanisms [27–29]. The connective tissue within the vaginal wall adjusts to biomechanical alterations via extracellular matrix (ECM) metabolism. This remodeling activity is highly dependent on the connective tissue′s cellular elements, with fibroblasts being the primary drivers [26]. The fibrillar component, which greatly impacts the biomechanical characteristics of pelvic tissues, is governed by a delicate balance between the synthesis, maturation, and breakdown of collagen and elastin. Fibroblasts are at the core of this regulatory process, dictating the fibrillar component′s quality and quantity [30–33]. Disruptions in ECM and collagen metabolism can be triggered by genetic mutations or decreased estrogen levels [30, 34]. These disruptions, such as a reduction in collagen quantity, an upsurge in collagen degradation, or modifications in ECM remodeling and structural protein expression, heighten the susceptibility to POP [35]. Considering that fibroblasts are critical for collagen metabolism and ECM turnover, any deviation in their function can have a profound effect on the emergence of POP. In individuals suffering from POP, the connective tissue undergoes remarkable changes in both quality and quantity [35]. Whether caused by genetic factors or defects acquired from mechanical stress, the function of fibroblasts in prolapsed tissue deviates from the norm. This functional disparity leads to modifications in collagen content, which is especially important as collagen makes up as much as 80% of the connective tissue [24]. Among the 28 collagen types, collagen type I and III are the most characteristic in the pelvic floor and are key determinants of tissue strength [36, 37]. Collagen I confers strong stretching and tensile resistance capabilities, strengthening the pelvic structures, while collagen III provides flexibility and extensibility, being abundant in tissues subjected to cyclic stress [37, 38]. The quantity of collagen in pelvic tissue and the ratio between collagen I and III mirror the tissue′s biomechanical properties, elasticity, and relaxation potential [33, 39, 40]. A higher I/III ratio is indicative of greater tissue strength, whereas a lower ratio points to tissue laxity [24]. Significantly, POP patients typically exhibit lower overall collagen content compared with those without the condition [35]. Additionally, the contractility of vaginal (myo) fibroblasts is diminished in POP patients, potentially resulting in collagen insufficiency [30]. Multiple studies have also demonstrated that, regardless of age and parity, the sub‐epithelium and muscular layer of the vaginal wall in POP patients have elevated collagen III levels compared to non‐POP women [33, 39, 40]. This increase in collagen III might signify an attempt at tissue repair after overstretching of the pelvic floor′s supportive connective tissue. However, because increased collagen III is linked to enhanced flexibility and extensibility but reduced tensile strength in pelvic tissues, it can also contribute to the advancement of POP. In conclusion, alterations in gene expression regulated by fibroblasts, particularly those associated with collagen metabolism, are likely crucial factors propelling the development of POP.

Our research findings indicate that *RBP7* is a risk factor for the onset of POP, while *SCGB3A1* is a protective factor for the development of POP. *RBP7* is a member of the cellular retinol‐binding protein (CRBP) family [41]. The protein encoded by the *RBP7* gene is essential for various BP, including cell proliferation, adipocyte differentiation, and apoptosis [42, 43]. *RBP7* is located on human Chromosome 1p36.22 and contains 134 amino acids [41]. RBP7 is involved in the metabolism of vitamin A and its derivatives, which are critical for maintaining cellular homeostasis and function [43]. Vitamin A and its metabolites play a crucial role in regulating the gene expression and synthesis of ECM components such as collagen [44, 45]. Retinoic acid (RA), as the major biologically active form of vitamin A, can regulate the transcription of genes related to collagen fibers by binding to nuclear receptors RARs (retinoic acid receptors) and RXRs (retinoid X receptors) [46–48]. RA has the ability to inhibit the expression of collagen type I chains (*α*1 (I) and *α*2 (I)) [49, 50]. The underlying mechanism lies in the binding of the RAR–RXR heterodimer to the retinoic acid response elements (RAREs) within the promoters of these genes, which consequently reduces the synthesis of collagen type I. Moreover, vitamin A also has an indirect impact on collagen fibers by influencing ECM receptors and related signaling pathways [48]. ECM receptors like integrins can interact with collagen fibers [51, 52]. A deficiency of vitamin A may alter the expression or function of these receptors, thereby affecting the interaction between collagen fibers and cells, as well as the regulation of collagen fiber metabolism by cells. In addition, vitamin A interacts with signaling pathways such as TGF‐*β*/Smad [47]. TGF‐*β*1 can regulate the expression of various collagens through the SMAD signaling pathway [53, 54]. When there is a deficiency of vitamin A, the level of TGF‐*β*1 increases, which may indirectly promote the synthesis and deposition of collagen fibers [48]. In patients with POP, the total collagen content in the pelvic floor tissues is significantly reduced, and the ratio of collagen I/III is altered [38, 55]. RBP7 may indirectly affect the function of fibroblasts in synthesizing and secreting collagen by maintaining a stable intracellular level of vitamin A. If the function of RBP7 is abnormal, it may lead to a decrease in the ability of fibroblasts to synthesize collagen, resulting in a reduction of the collagen content in the pelvic floor tissues and a change in the collagen I/III ratio. This, in turn, affects the mechanical properties of the pelvic floor tissues and increases the risk of developing POP. In the data set validation of the Japanese cohort, *RBP7* was also confirmed as a possible risk factor for POP.

*SCGB3A1* is a protein‐coding gene located on Chromosome 5q35.3 which is responsible for encoding a small secretory protein [56, 57]. It is highly expressed in the epithelial cells of normal lung, uterus, prostate, and breast [56, 58]. *SCGB3A1* is involved in regulating epithelial cell proliferation, differentiation, and morphogenesis [59, 60]. Abnormal expression of *SCGB3A1* has been associated with the development of malignant phenotypes in human tumors [61, 62]. Currently, the role of SCGB3A1 in fibroblasts has not been reported yet. Most of the relevant studies on SCGB3A1 have been concentrated on respiratory diseases [63–65]. SCGB1A1, which is abundantly present in the airway lining fluid, has a complex function. It acts not only as a biomarker for chronic obstructive pulmonary disease (COPD) injury but also plays a part in the anti‐inflammatory process [66–70]. As reported, SCGB1A1 exerts its anti‐inflammatory effects through multiple mechanisms, such as inhibiting the chemotaxis of inflammatory cells and regulating cytokines [71–73], and is associated with both innate immunity and adaptive immunity [74–77]. We hypothesize that SCGB3A1 might perform similar functions in fibroblasts, reducing the chemotactic aggregation of inflammatory cells like neutrophils and macrophages towards the pelvic floor tissues. The excessive accumulation of these inflammatory cells will result in the release of a variety of inflammatory mediators, which can damage the ECM of the pelvic floor tissues and interfere with the normal functions of fibroblasts. By suppressing the chemotaxis of inflammatory cells, SCGB3A1 can mitigate the inflammatory microenvironment of the pelvic floor tissues, safeguard fibroblasts and the ECM, thus decreasing the risk of the development of POP. Furthermore, SCGB3A1 may, just like SCGB1A1, be involved in regulating the secretion of cytokines by fibroblasts [75]. It is probable that it inhibits the secretion of pro‐inflammatory cytokines (such as tumor necrosis factor‐*α* and interleukin‐6) while promoting the production of anti‐inflammatory cytokines (such as interleukin‐10). This balanced regulation of cytokines contributes to alleviating the inflammatory response in the pelvic floor tissues, maintaining the normal metabolism and functions of fibroblasts, enabling them to synthesize and secrete the ECM appropriately and preserve the supporting structure of the pelvic floor tissues. The lack of significant *SCGB3A1* associations with POP in the Japanese cohort does not negate its protective role in our European population, potentially due to population‐specific genetic or regulatory differences. In the future, additional research is required to clarify the regulatory role and mechanism of *SCGB3A1* in POP and validate across larger trans‐ethnic cohorts.

In addition, we have conducted some explorations on potential therapeutic drugs for POP. Our research findings indicate that benzo(a)pyrene, estradiol, fenretinide, diethylhexyl phthalate, and 3,4,5,3′,4′‐pentachlorobiphenyl may have the potential to target *RBP7* for the treatment of POP. Meanwhile, benzo(k)fluoranthene, perfluorooctane sulfonic acid, triptonide, aflatoxin B1, and doxorubicin may have the potential to target *SCGB3A1* for POP treatment. Among these candidate drugs, due to toxicity issues, benzo(a)pyrene, diethylhexyl phthalate, 3,4,5,3′,4′‐pentachlorobiphenyl, benzo(k)fluoranthene, perfluorooctane sulfonic acid, and aflatoxin B1 are not feasible for the treatment of POP. Theoretically speaking, estrogen holds the potential to treat POP. Estrogen, a key female sex hormone, plays diverse roles in maintaining the integrity and function of pelvic tissues. In the context of POP, which is characterized by the descent and protrusion of pelvic organs, estrogen′s potential therapeutic action is hypothesized to be based on its ability to enhance the strength and elasticity of the pelvic floor muscles and connective tissues. It may also influence cell‐level processes such as collagen synthesis and cell proliferation within these tissues. Some in vitro studies have provided preliminary evidence, showing that estrogen can promote the growth and function of relevant cells in the pelvic floor, thereby suggesting a possible inhibitory effect on the progression of POP [78]. However, despite these theoretical and initial experimental indications, the actual application of estrogen in the treatment of POP remains a subject of intense research. Clinical trials so far have not been able to fully validate its significant efficacy, likely due to various factors such as differences in patient populations, dosing regimens, and treatment durations [79–81]. Thus, further comprehensive studies are urgently needed to fully explore and clarify estrogen′s role in POP treatment. Currently, there are no studies on the use of fenretinide for the treatment of POP, and its feasibility remains to be further explored. Theoretically, as a retinoid drug [82], it may have a certain regulatory effect on cell differentiation and [83–86], thereby influencing the repair and reconstruction of the pelvic floor tissues. However, this needs to be confirmed by more experimental and clinical studies. Fenretinide may affect the activity of fibroblasts and the synthesis and degradation of collagen by regulating the intracellular signal transduction pathways [87, 88]. Studies have shown that triptonide has immunomodulatory and anti‐inflammatory effects in some inflammation‐related diseases [89–91], and the occurrence of POP is associated with the chronic inflammation of the pelvic floor tissues [92, 93]. Therefore, triptonide has certain theoretical feasibility in the treatment of POP, but further clinical studies are required for verification. Triptonide may reduce the release of inflammatory cytokines (such as tumor necrosis factor‐*α* and interleukin‐6) by inhibiting inflammatory signaling pathways such as nuclear factor‐*κ*B (NF‐*κ*B) [94–97], alleviating the inflammatory response of the pelvic floor tissues. Meanwhile, it may also regulate the function of immune cells and inhibit excessive immune responses [98], thus protecting the pelvic floor tissues, helping to maintain the normal structure and function of the pelvic floor tissues, and reducing the risk of POP. doxorubicin is mainly used for the treatment of cancer [99]. Although POP is related to cell proliferation and tissue remodeling, doxorubicin is not a drug specifically designed for the treatment of POP. Currently, there is insufficient evidence to support its routine application in the treatment of POP, and its feasibility needs further research. doxorubicin inhibits cell proliferation by intercalating into the double strands of DNA and inhibiting the activity of topoisomerase II, thus preventing DNA replication and transcription [100]. In the pelvic floor tissues, it may have a certain inhibitory effect on excessively proliferating cells (such as fibroblasts in some pathological states), but it will also be toxic to normal cells. In addition, doxorubicin may also indirectly affect the repair and reconstruction process of the pelvic floor tissues by influencing the secretion of cytokines and signal transduction, but these effects need to be further clarified through more research. It is recommended that subsequent research focus on evaluating the therapeutic effects and mechanisms of estradiol, fenretinide, triptonide, and doxorubicin on POP.

Despite the significant insights provided by our innovative integration of single‐cell sequencing data analysis and MR analysis to identify KGs like *RBP7* and *SCGB3A1* in POP pathogenesis and explore potential therapeutic drugs, our study has several limitations. The single‐cell sequencing data might not comprehensively cover all fibroblast populations relevant to POP, and the MR analysis relied on existing eQTL data which could be incomplete or inaccurate. Our understanding of the role of *SCGB3A1* in fibroblasts mainly stems from speculation based on previous research data, yet it lacks direct experimental validation. For the potential therapeutic drugs, the in vitro and in vivo studies on their effects on POP are scarce, especially for fenretinide, triptonide, and doxorubicin. In the future, we should conduct more in‐depth single–cell sequencing studies to fully capture fibroblast heterogeneity in POP, experimentally validate the functions of *SCGB3A1* in fibroblasts, and perform extensive preclinical trials on the candidate drugs to evaluate their safety and efficacy. Overall, our study has laid a foundation by identifying crucial genes and potential drugs related to POP, but much work remains to be done to translate these findings into effective clinical treatments for POP patients.

## 5. Conclusion

In conclusion, this study, through integrating single‐cell sequencing and MR analysis, identified *RBP7* as a POP risk factor and *SCGB3A1* as a protective one, highlighting the significance of fibroblast gene expression changes in collagen metabolism for POP progression. We explored potential drugs: Estradiol shows potential in treating postmenopausal POP patients by improving collagen metabolism, whereas Fenretinide, Triptonide, and Doxorubicin need further study but have theoretical potential to regulate fibroblast functions. Future research should experimentally verify SCGB3A1′s functions in fibroblasts, conduct preclinical drug trials, and use better data and techniques to clarify POP′s molecular mechanisms for more effective treatment strategies.

## Ethics Statement

The authors have nothing to report.

## Disclosure

All authors perused the final manuscript and provided their consent.

## Conflicts of Interest

The authors declare no conflicts of interest.

## Author Contributions

The research design was crafted by Y.Y., F.D., and R.C. Data collection was executed by Y.Y., W.X., X.W., and F.D. R.C. and Q.S. were tasked with analyzing and interpreting the data. N.H. and W.X. undertook the literature search. Y.Y. and R.J.C. penned the initial version of the article. R.C. and L.Z. took charge of supervision, obtaining funding, and manuscript review. Y.Y., and W.X. contributed equally to this work.

## Funding

This study was supported by Shanghai Fifth People′s Hospital, Fudan University (2024WYRCJY03); High‐level Professional Physician Training Program of Minhang District, Shanghai (2024MZYS15).

## Supporting Information

Additional supporting information can be found online in the Supporting Information section.

## Supporting information


**Supporting Information 1** Table S1. The list of differentially expressed genes in fibroblasts between the POP group and the non‐POP group.

**Supporting Information 2** Figure S1. Forest plot demonstrates the association of *RBP7*/*SCGB3A1* SNPs with POP in validation data.

**Supporting Information 3** Table S2. The list of the binding probabilities of candidate therapeutic drugs to RBP7.

**Supporting Information 4** Table S3. The list of the binding probabilities of candidate therapeutic drugs to SCGB3A1.

**Supporting Information 5** Figure S2. Manhattan plot demonstrates the association of *RBP7*/*SCGB3A1* SNPs with POP in validation data.

## Data Availability

The data that support the findings of this study are available from the corresponding author upon reasonable request.
