# Supplementary figures and images for: Unraveling the Causal Linkages of RBP7 and SCGB3A1 on Pelvic Organ Prolapse: Multifaceted Insights From Genome‐Wide Mendelian Randomization, Single‐Cell RNA Analysis, and Network Pharmacology
Source: Biomed Res Int. 2026 Jan 4;2026:9785848. doi: 10.1155/bmri/9785848 (PMC12765987; doi:10.1155/bmri/9785848)

Association of RBP7/SCGB3A1 SNPs with POP in Validation Data

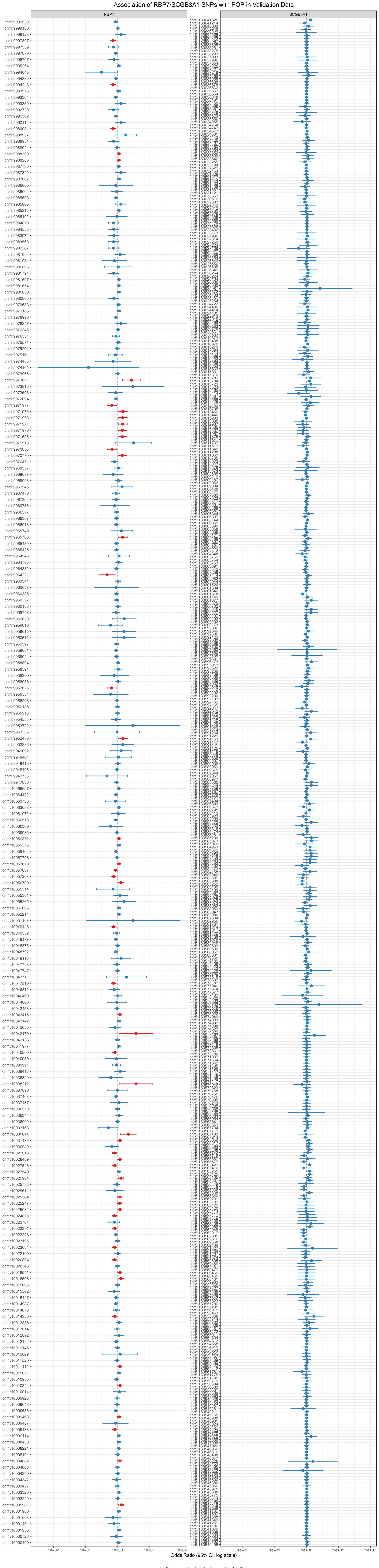

Supplement: Supplementary file 2 — Supporting Information 2 Figure S1. Forest plot demonstrates the association of RBP7/SCGB3A1 SNPs with POP in validation data. [file BMRI-2026-9785848-s005.pdf]

Manhattan Plot of SNPs in RBP7/SCGB3A1 Regions

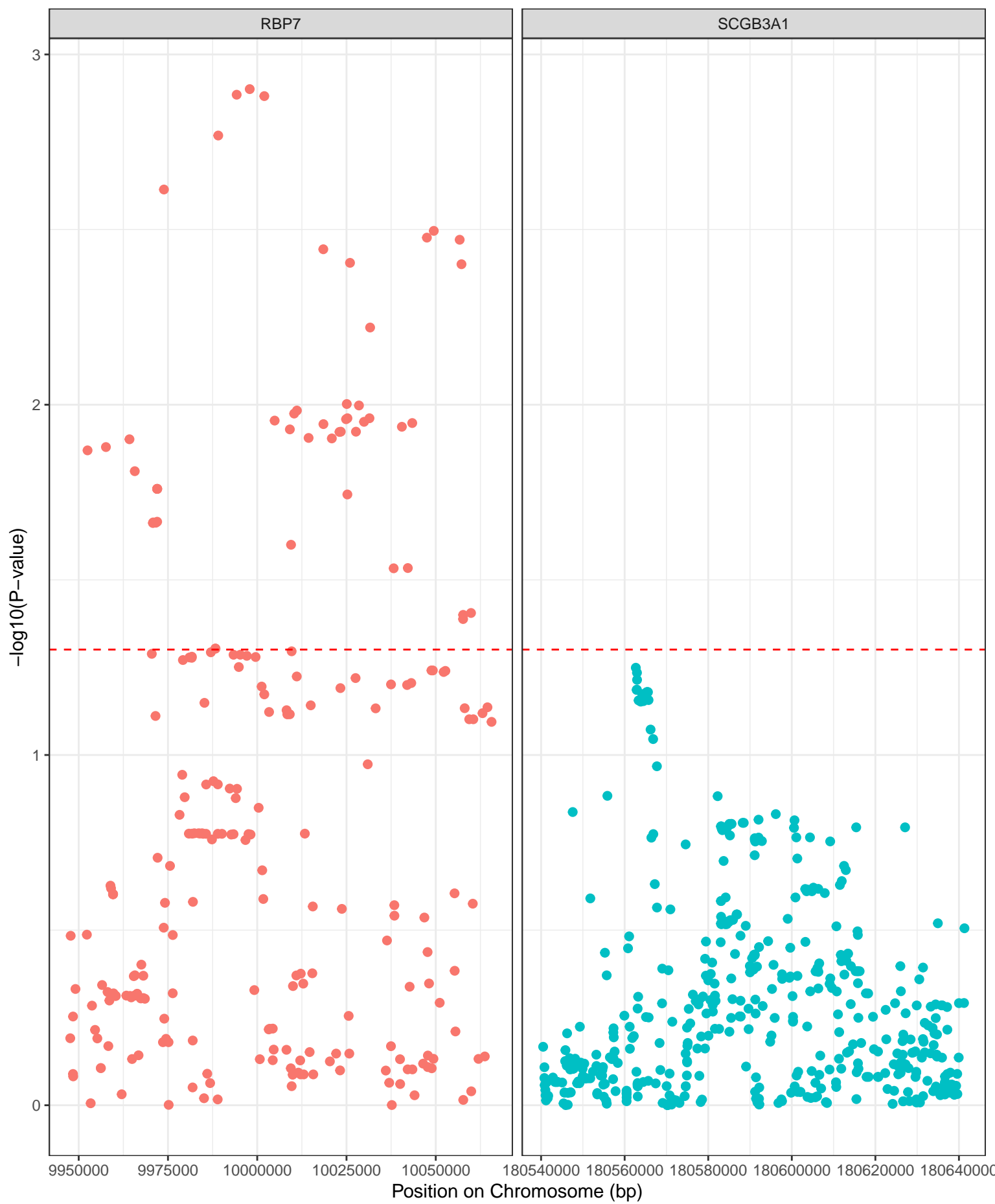

Supplement: Supplementary file 5 — Supporting Information 5 Figure S2. Manhattan plot demonstrates the association of RBP7/SCGB3A1 SNPs with POP in validation data. [file BMRI-2026-9785848-s001.pdf]
